# Supplementary material for: Antibiotic residues and antibiotic-resistant bacteria detected in milk marketed for human consumption in Kibera, Nairobi
Source: PLoS One. 2020 May 28;15(5):e0233413. doi: 10.1371/journal.pone.0233413 (PMC7255607; doi:10.1371/journal.pone.0233413)
Supplement: Attachment — Submitted filename: Response to Reviewers.docx [file pone.0233413.s003.docx]

**Comment 1**

We understand that you purchased milk from local markets for this study. In your Methods section, please provide additional details regarding the source of this material.

- Please provide the geographic coordinates and names of the purchase locations (e.g., shops, vendors), if available.

>> We have provided a study description for this community, but we cannot supply the requested geographic coordinates. This is because such coordinates are not available for the mobile vendors who sell raw milk or even for the formal vendors that typically erect a shop that is later moved or abandoned within a matter of months. As such, reporting such coordinates would not help in the replication of this study

- Further details about the purchased items (e.g., quantity, source origin) to ensure reproducibility of the analyses.

>> These details have now been included. We have also included a figure (not Fig 1) to illustrate the packaging of these milk samples. We did not collect data regarding the source origin of the milk samples we purchased.

**Comment 2**

Thank you for including the following funding information within the acknowledgements section of your manuscript; "This work was funded by the Paul G. Allen School for Global Animal Health, and by the Washington State University College of Veterinary Medicine Summer Research Fellowship Program. "

- Please remove any funding-related text from the manuscript and let us know how you would like to update your Funding Statement. Currently, your Funding Statement reads as follows: "The funders had no role in study design, data collection and analysis, decision to publish, or preparation of the manuscript."

>> Funding information deleted from the acknowledgement section

**Comment 3**

The data should be provided as part of the manuscript or its supporting information, or deposited to a public repository. For example, in addition to summary statistics, the data points behind means, medians and variance measures should be available. If there are restrictions on publicly sharing data—e.g. participant privacy or use of data from a third party—those must be specified.

>> The dataset has been provided as part of supporting information.

**Comment 4**

The reviewers observe merit in the study, but there are significant concerns relative to the methods presented and the statistical analyses conducted.

- The authors need to clearly state how the study was conducted and the analyses that were done.

>> Specific questions about the methods and analyses have been clarified in the revision as indicated under each specific comment below.

- Furthermore, the data presentation could be improved, and statistical analyses need to be conducted properly as noted by Reviewer 1.
  >> We believe that we have addressed the clarity issues that were noted by the reviewer. Please note that the reviewer mistakenly concluded that we calculated the proportion of resistant bacteria by making a ratio of the CFU count from agar plates without antibiotic and agar plates with antibiotic. All our comparisons are on a sample basis (presence or absence of detectable antibiotic-resistant bacteria) as the reviewer suggests should have been done. We report total counts from the plates, but not a proportion of the plate without antibiotic. It is unclear to us why this confusion may have occurred.

**Comment 5**
Reviewer #1: The manuscript by Brown et al presents results of public health importance. They reported antibiotic residues in 10% of ready to consume milk, and majority of unpasteurized milk samples containing high bacterial load including antibiotic resistant bacteria.

- Although the study is of great interest, the methods are vaguely presented, results are inconsistent, poorly presented and discussed. Please see general and specific comments below.
- Abstract
  - Line 32: please change “hypoallergenic” to “hyperallergenic” as used in the body of the manuscript (line 61). I suggest using the more general term (without the specific type) “allergic”.

>> we have replaced this term with “immunogenic”

- Line 43-44 and elsewhere in the text: would you present the CFU/ml values in log10 scale?

>> This has now been addressed.

- Line 43-44 and elsewhere in the text: how these percentages (note that in the text it was mentioned 92% for ampicillin line 131) were calculated? Please clearly state this in the materials and methods.

>> The calculation of all percentages has been clarified by indicating the specific counts that form the numerators and denominators.

- How is it possible to calculate resistant fraction obtained on media supplemented with antibiotics from total bacterial count obtained from plain media? This biases towards the numerator as we already select for the resistant population, simply the methods by which the two values were determined are different. For the stated proportions replica platting would be appropriate. See more comment on this below.

>>As above, we are not sure why the reviewer drew this conclusion about the methods. There is no text describing calculation of a resistant fraction in terms of bacterial numbers. All proportion values that we report are relative to the proportion of positive or negative samples, not bacterial counts. For bacterial counts, we calculated total bacterial counts based on the corresponding plates from which these counts were made.

- Line 45: How did you arrive at the conclusion of “no evidence”? Please show statistical analysis.

>> We have revised this statement to read: “We found no relationship between the presence of antibiotic residues and the abundance of antibiotic-resistant *E. coli*”.

- Introduction
  - Please add literature (perhaps between lines 61 and 62) on the maximum residue level (MRL) allowed in milk for the beta-lactam and oxytetracycline.

>> This information has been added

- Line 71: replace” with” by “which”

>> Corrected. Thank you.

- Materials and methods
  - Line 89: Please add manufacturer’s information (company, city, State or Country)

>> This information has been added

- Line 99-100: what dilution(s) were plated? This is important since you used direct plating, too numerous to count indicates high bacterial load; on the other hand, most samples can be BDL if contamination is low which should be expected in milk (and water) and normally filtration is also used for detection.

>> The dilutions that were plated have been included in the revision.

- Line 113: please correct the degree sign as 37°C

>> This has been corrected. Thank you.

- Why your analytical sensitivity of 650 CFU/ml is high?

>> We agree that this value is higher than what would be expected with, for example, water, but it is an empirically derived value. We surmise that the count seems high because bacteria clump with milk components and thus a single colony from a milk sample potentially represents more than one bacterium. For the same reason, filtration would not have remedied this issue. Please note that if such clumping occurred, we consider this effect to be similar across all samples

- How many colonies per plate was your cut-off to count?

>> We primarily chose the dilution that gave us at least 10 colonies per plate. Counts below this threshold were only included for undiluted/neat milk samples. These thresholds have been included in the methods.

- Did you count 1CFU/plate and consider such a sample as positive, or how did you deal with? Please clarify your methods.

>> There was only one instance where one colony was observed in plate containing an undiluted milk sample. This plate had 5 colonies in the Mac agar without antibiotics, 1 in the Amp+ plate and no colonies in the Tet+ plate.

- Data analysis
  - Was the “random number” substitution a form of imputation? Please state.

>> This was based on a random number drawn from a uniform distribution. This has been added to the manuscript.

- Also discuss if this method would introduce a bias, for not adding it to observations with enumerable values.

>> We used this method to avoid issues that arise when transforming small numbers to a log scale (negative axis values) and because it acknowledges that we should not treat a negative value as truly negative given that there is a minimum analytic sensitivity of this assay. Not adding this value has no consequence to statistical comparisons that are made on a sample basis (positive vs. negative). Failure to add a similar randomly selected number between 0 and 650 would potentially bias the final counts to a lower value, but this is unlikely to be significant as the counts increase on a log scale.

- What log scale was used? If log10 state so.

>> This has now been addressed.

- Results
  - Line 129-130: please provide statistical significance for the difference.

>> This information has been added

- Line 130-131: as commented above please clarify how the percentages were calculated.
  “Among unpasteurized samples, 14 samples (67%) contained presumptive E. coli colonies out of which 92.8% and 50% were positive for AmpR– and TetR–E. coli, respectively.” This is inconsistent with what was presented in the abstract: Line 42-44: “One third of unpasteurized samples (8/21, 38%) contained detectable numbers of presumptive E. coli (mean 9.2 x 106 CFU/ml) and of these, 87% were positive for ampicillin- (3.7 x 106 CFU/ml) and 50% for tetracycline-resistant E. coli (1.4 x 106/ml).”

>>This was an error that has now been corrected.

- Line 130: what does the phrase “contained presumptive” indicate and how was it derived? Is it enumeration positive (i.e. above BDL)? Please clarify.

>>Please recall that we identified E. coli based only on colony morphology. While this works the majority of the time (cite your JMM paper), it is not perfect. We use “presumptive” as a qualifier to acknowledge this uncertainty.

- Please mention that E. coli (wild or resistant strains) were not observed on enumeration plate or are BDL.

>> This has now been addressed.

- Line 133-134: please also include the mean values (preferably log10) for each media type used.

>> This information has now been added.

- Line 136: given you have only tested two antibiotic classes, I would replace “multidrug-resistance” with co-selection.

>> This has been amended to make it clear that there is a probability that these were multi-drug resistant strains, even though we did not test for other resistance types. Additionally, we have moved this bit of statement to the discussion section to make it clear that this is our interpretation of this finding.

- Line 137: change “residuals” to residues.

>> This has been corrected. Thank you

- Fig: Please use log10 scale since most studies report that way.

>> This has now been addressed.

- In the title please add “by media type” to avoid confusion with drug residues.

>> We have changed values to log scale. We changed the title by inserting the word “detected” to eliminate confusion (although we are not certain that we follow the reviewer’s meaning with respect to the original title).

- For the milk samples with no plot observations (example #2, 7, 17-21), were the three types of bacteria (generic, Amp resistant and Tet resistant E. coli) absent across the three media types? Please state.

>> This information has been included in the figure caption.

- What do ME and MD mean? Please state.

>> This information has been included in the figure caption.

- Discussion
  - Line 146: please insert “unpasteurized” before “milk” and elsewhere in the discussion. Pasteurization removes this issue.

>> This has been amended as suggested.

- Line 146-148: again, you did not test individual colonies of the total E. coli you obtained on plain media to give you proportion resistant, which would only be possible through replica platting. Rather you isolated the three bacterial strains independently. So, present all your results on sample basis throughout the manuscript.

>> we are uncertain why the reviewer is making this argument since our results are presented on a sample basis. We report total counts with and without antibiotic in the agar, but we did not calculate a proportion of resistant bacteria based on these values.

- Line 161: should read “represent”

>> This has been corrected. Thank you
